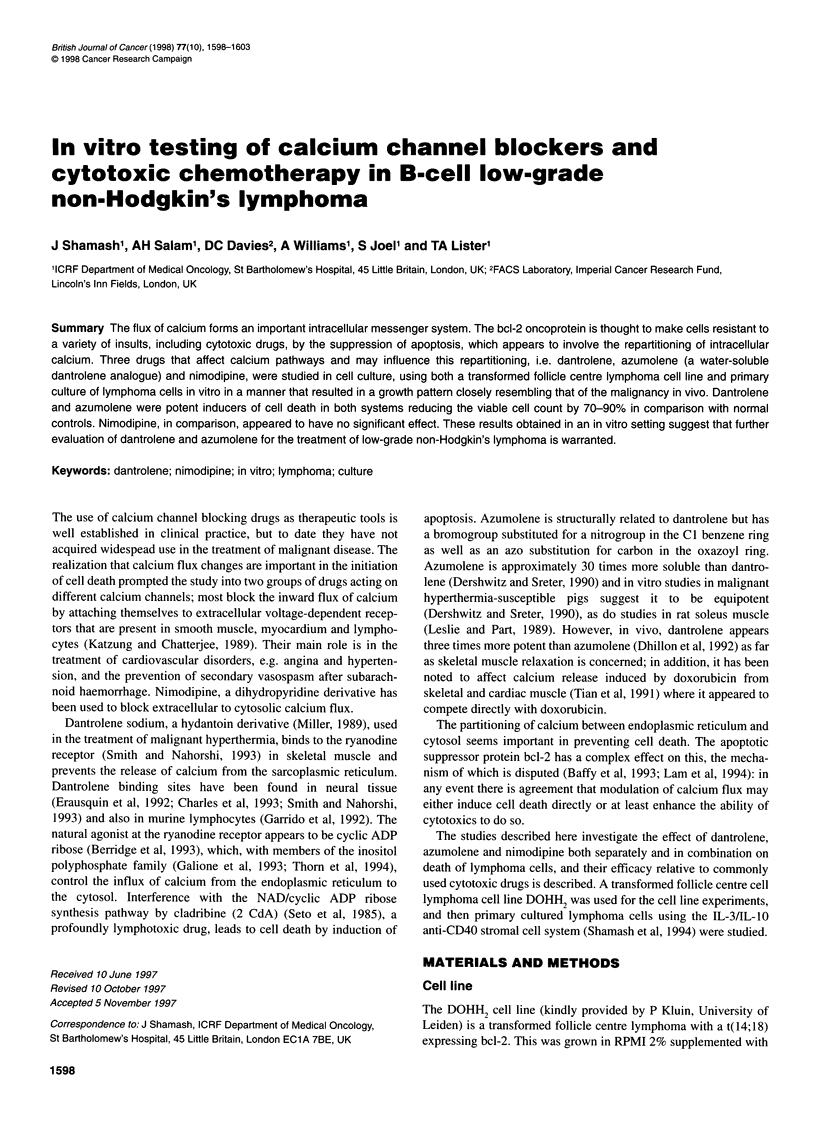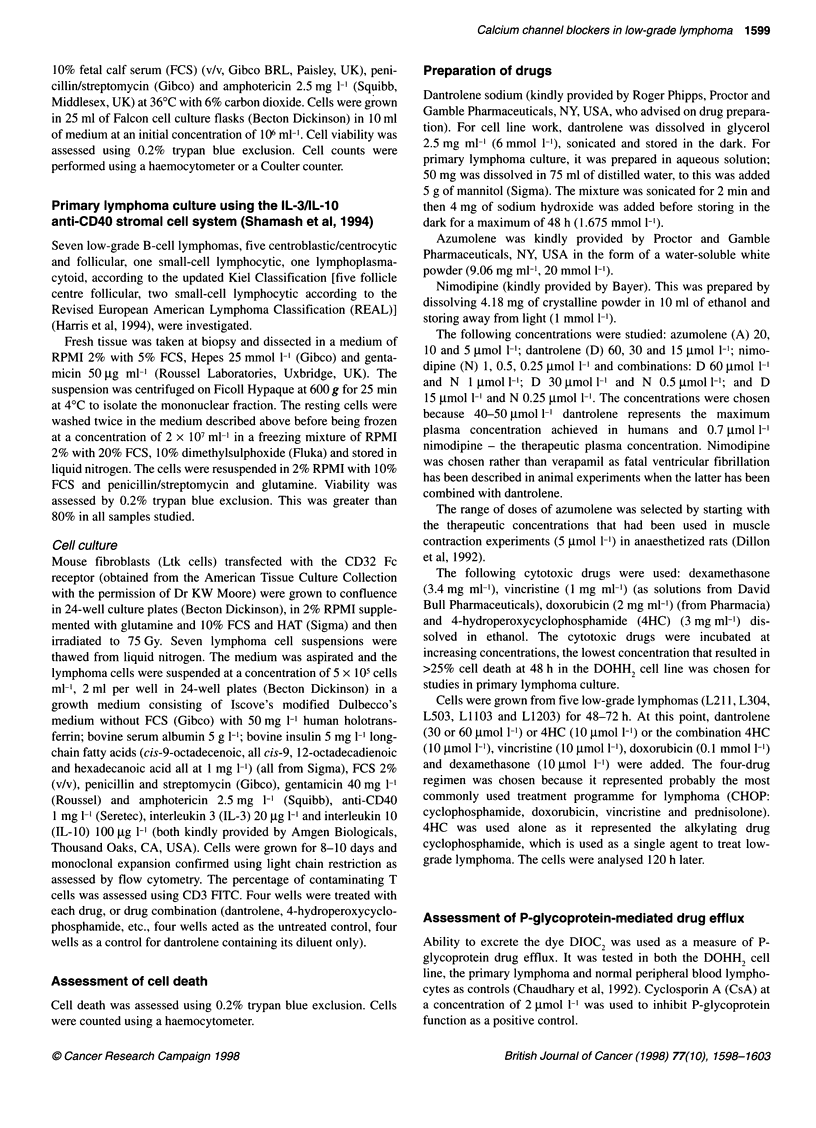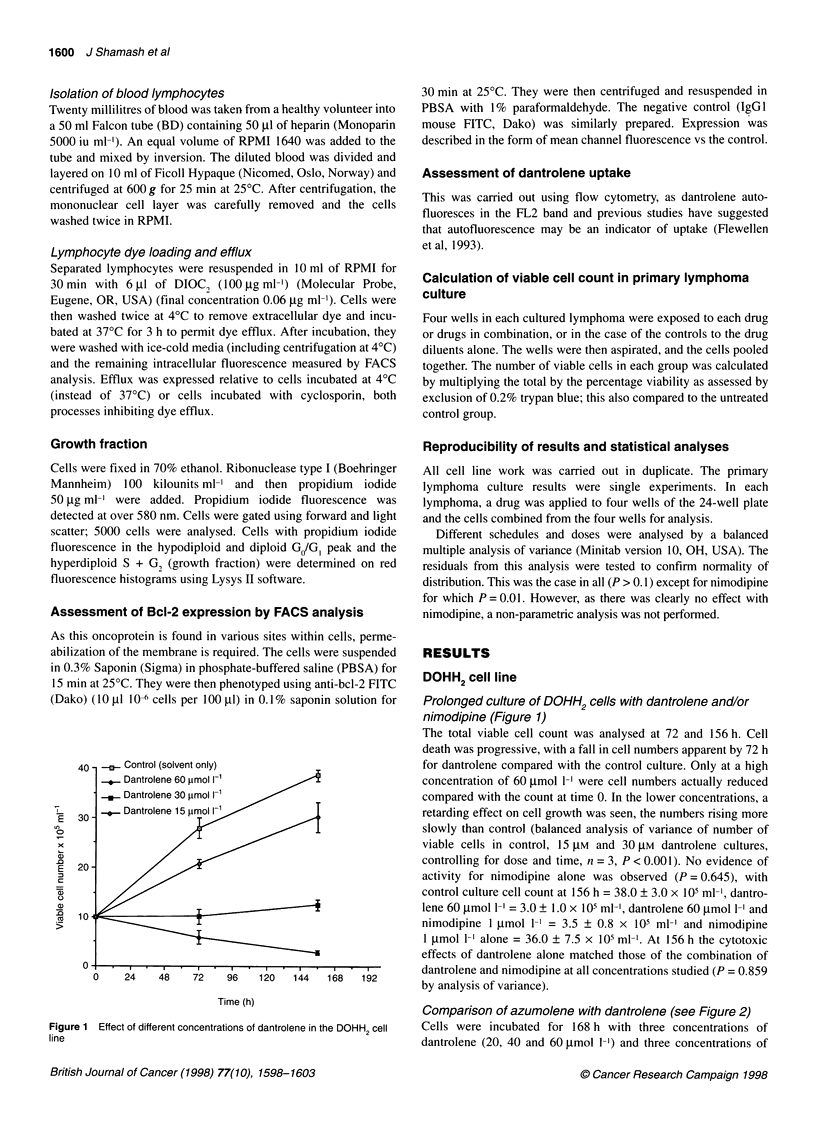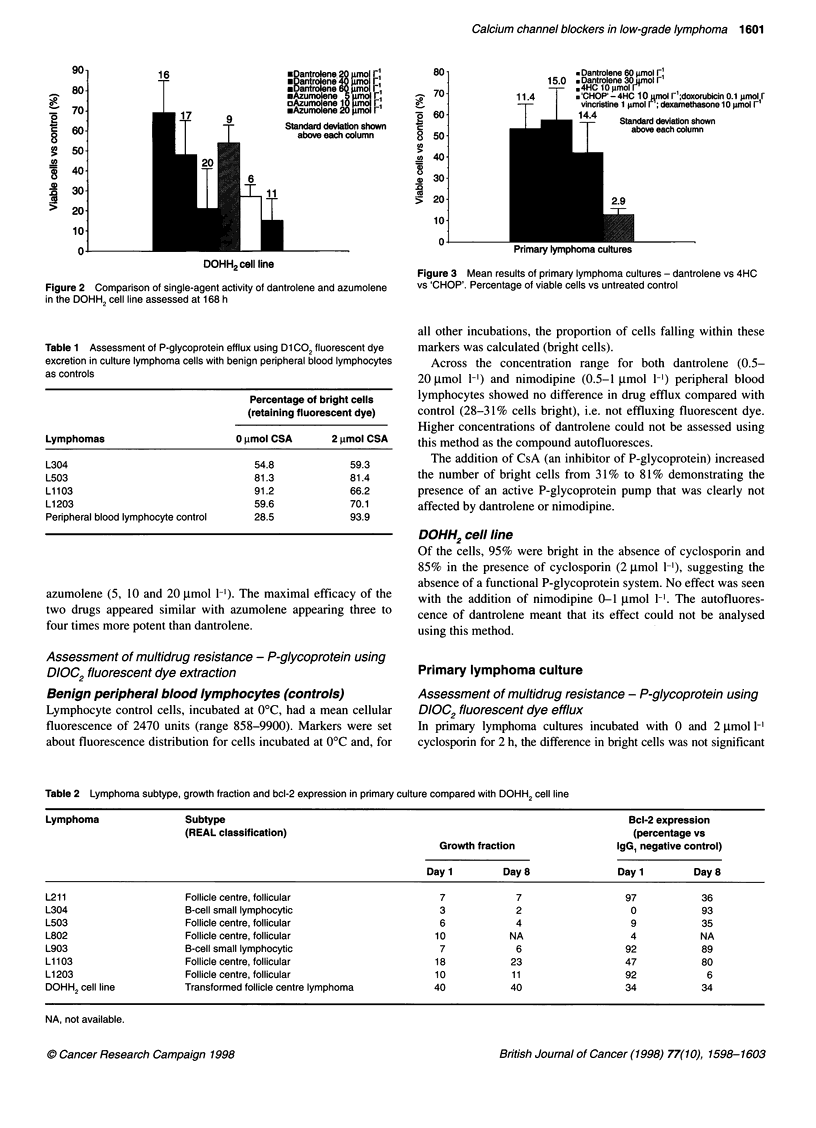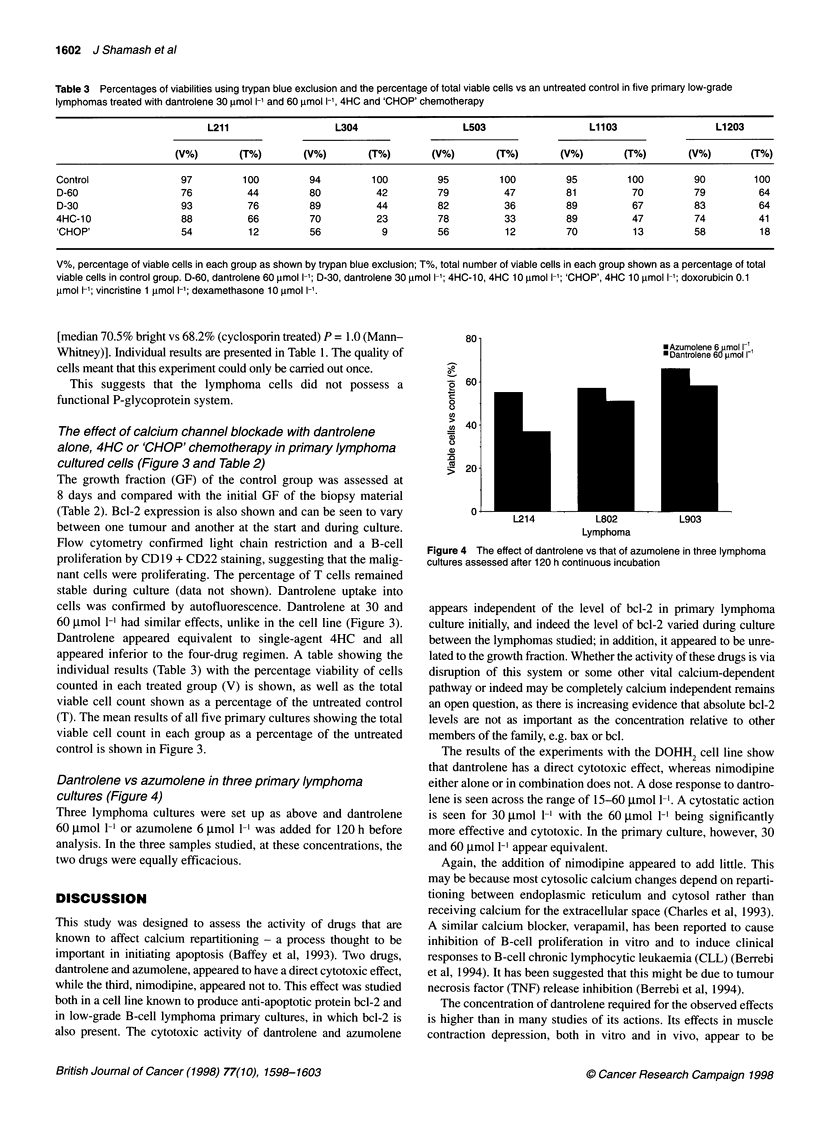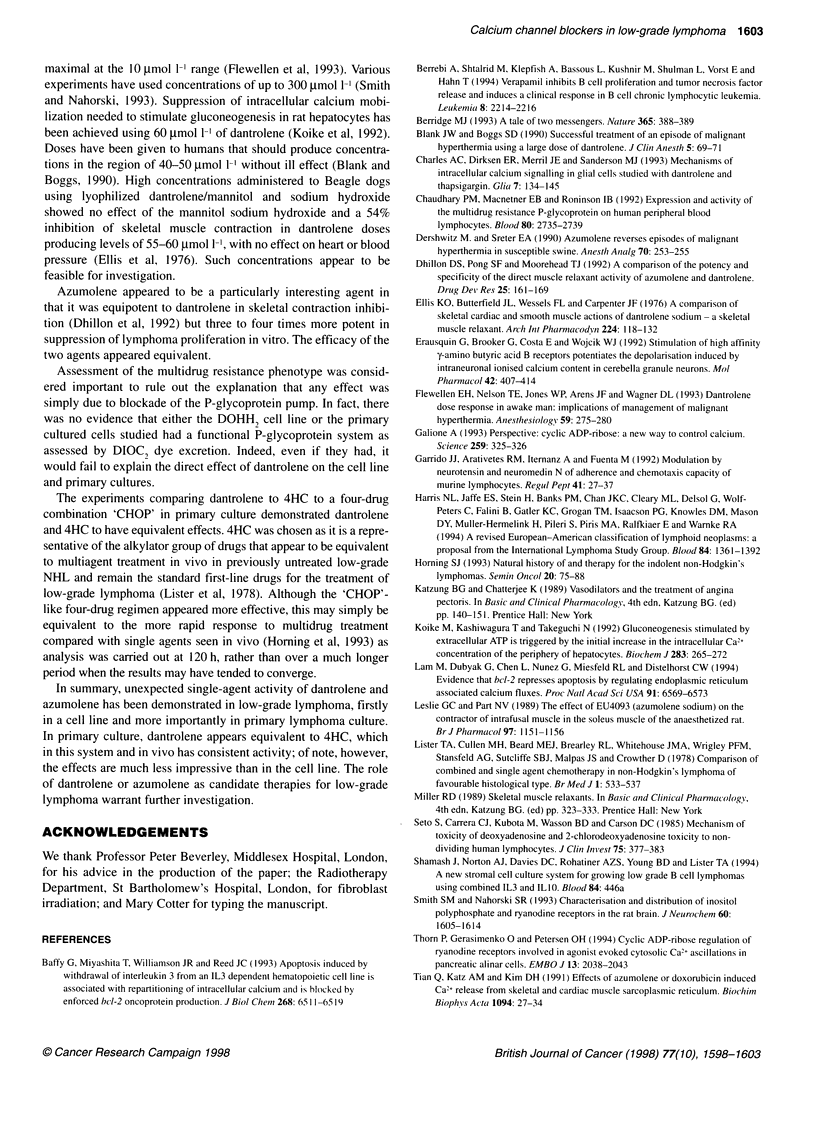# In vitro testing of calcium channel blockers and cytotoxic chemotherapy in B-cell low-grade non-Hodgkin's lymphoma.

**DOI:** 10.1038/bjc.1998.262

**Published:** 1998-05

**Authors:** J. Shamash, A. H. Salam, D. C. Davies, A. Williams, S. Joel, T. A. Lister

**Affiliations:** ICRF Department of Medical Oncology, St Bartholomew's Hospital, London, UK.

## Abstract

The flux of calcium forms an important intracellular messenger system. The bcl-2 oncoprotein is thought to make cells resistant to a variety of insults, including cytotoxic drugs, by the suppression of apoptosis, which appears to involve the repartitioning of intracellular calcium. Three drugs that affect calcium pathways and may influence this repartitioning, i.e. dantrolene, azumolene (a water-soluble dantrolene analogue) and nimodipine, were studied in cell culture, using both a transformed follicle centre lymphoma cell line and primary culture of lymphoma cells in vitro in a manner that resulted in a growth pattern closely resembling that of the malignancy in vivo. Dantrolene and azumolene were potent inducers of cell death in both systems reducing the viable cell count by 70-90% in comparison with normal controls. Nimodipine, in comparison, appeared to have no significant effect. These results obtained in an in vitro setting suggest that further evaluation of dantrolene and azumolene for the treatment of low-grade non-Hodgkin's lymphoma is warranted.


British Journal of Cancer (1998) 77(10), 1598–1603
© 1998 Cancer Research Campaign

### In vitro testing of calcium channel blockers and cytotoxic chemotherapy in B-cell low-grade non-Hodgkin's lymphoma

**J Shamash[1], AH Salam[1], DC Davies[2], A Williams[1], S Joel[1] and TA Lister[1]**

[1]ICRF Department of Medical Oncology, St Bartholomew's Hospital, 45 Little Britain, London, UK; [2]FACS Laboratory, Imperial Cancer Research Fund, Lincoln's Inn Fields, London, UK

**Summary** The flux of calcium forms an important intracellular messenger system. The bcl-2 oncoprotein is thought to make cells resistant to a variety of insults, including cytotoxic drugs, by the suppression of apoptosis, which appears to involve the repartitioning of intracellular calcium. Three drugs that affect calcium pathways and may influence this repartitioning, i.e. dantrolene, azumolene (a water-soluble dantrolene analogue) and nimodipine, were studied in cell culture, using both a transformed follicle centre lymphoma cell line and primary culture of lymphoma cells in vitro in a manner that resulted in a growth pattern closely resembling that of the malignancy in vivo. Dantrolene and azumolene were potent inducers of cell death in both systems reducing the viable cell count by 70–90% in comparison with normal controls. Nimodipine, in comparison, appeared to have no significant effect. These results obtained in an in vitro setting suggest that further evaluation of dantrolene and azumolene for the treatment of low-grade non-Hodgkin's lymphoma is warranted.

**Keywords:** dantrolene; nimodipine; in vitro; lymphoma; culture

The use of calcium channel blocking drugs as therapeutic tools is well established in clinical practice, but to date they have not acquired widespead use in the treatment of malignant disease. The realization that calcium flux changes are important in the initiation of cell death prompted the study into two groups of drugs acting on different calcium channels; most block the inward flux of calcium by attaching themselves to extracellular voltage-dependent receptors that are present in smooth muscle, myocardium and lymphocytes (Katzung and Chatterjee, 1989). Their main role is in the treatment of cardiovascular disorders, e.g. angina and hypertension, and the prevention of secondary vasospasm after subarachnoid haemorrhage. Nimodipine, a dihydropyridine derivative has been used to block extracellular to cytosolic calcium flux.

Dantrolene sodium, a hydantoin derivative (Miller, 1989), used in the treatment of malignant hyperthermia, binds to the ryanodine receptor (Smith and Nahorshi, 1993) in skeletal muscle and prevents the release of calcium from the sarcoplasmic reticulum. Dantrolene binding sites have been found in neural tissue (Erausquin et al, 1992; Charles et al, 1993; Smith and Nahorshi, 1993) and also in murine lymphocytes (Garrido et al, 1992). The natural agonist at the ryanodine receptor appears to be cyclic ADP ribose (Berridge et al, 1993), which, with members of the inositol polyphosphate family (Galione et al, 1993; Thorn et al, 1994), control the influx of calcium from the endoplasmic reticulum to the cytosol. Interference with the NAD/cyclic ADP ribose synthesis pathway by cladribine (2 CdA) (Seto et al, 1985), a profoundly lymphotoxic drug, leads to cell death by induction of apoptosis. Azumolene is structurally related to dantrolene but has a bromogroup substituted for a nitrogroup in the C1 benzene ring as well as an azo substitution for carbon in the oxazoyl ring. Azumolene is approximately 30 times more soluble than dantrolene (Dershwitz and Sreter, 1990) and in vitro studies in malignant hyperthermia-susceptible pigs suggest it to be equipotent (Dershwitz and Sreter, 1990), as do studies in rat soleus muscle (Leslie and Part, 1989). However, in vivo, dantrolene appears three times more potent than azumolene (Dhillon et al, 1992) as far as skeletal muscle relaxation is concerned; in addition, it has been noted to affect calcium release induced by doxorubicin from skeletal and cardiac muscle (Tian et al, 1991) where it appeared to compete directly with doxorubicin.

The partitioning of calcium between endoplasmic reticulum and cytosol seems important in preventing cell death. The apoptotic suppressor protein bcl-2 has a complex effect on this, the mechanism of which is disputed (Baffy et al, 1993; Lam et al, 1994): in any event there is agreement that modulation of calcium flux may either induce cell death directly or at least enhance the ability of cytotoxics to do so.

The studies described here investigate the effect of dantrolene, azumolene and nimodipine both separately and in combination on death of lymphoma cells, and their efficacy relative to commonly used cytotoxic drugs is described. A transformed follicle centre cell lymphoma cell line $DOHH_2$ was used for the cell line experiments, and then primary cultured lymphoma cells using the IL-3/IL-10 anti-CD40 stromal cell system (Shamash et al, 1994) were studied.

### MATERIALS AND METHODS

### Cell line

The $DOHH_2$ cell line (kindly provided by P Kluin, University of Leiden) is a transformed follicle centre lymphoma with a t(14;18) expressing bcl-2. This was grown in RPMI 2% supplemented with

*Received 10 June 1997*
*Revised 10 October 1997*
*Accepted 5 November 1997*

*Correspondence to:* J Shamash, ICRF Department of Medical Oncology, St Bartholomew's Hospital, 45 Little Britain, London EC1A 7BE, UK

10% fetal calf serum (FCS) (v/v, Gibco BRL, Paisley, UK), penicillin/streptomycin (Gibco) and amphotericin 2.5 mg $l^{-1}$ (Squibb, Middlesex, UK) at 36°C with 6% carbon dioxide. Cells were grown in 25 ml of Falcon cell culture flasks (Becton Dickinson) in 10 ml of medium at an initial concentration of $10^6$ $ml^{-1}$. Cell viability was assessed using 0.2% trypan blue exclusion. Cell counts were performed using a haemocytometer or a Coulter counter.

### Primary lymphoma culture using the IL-3/IL-10 anti-CD40 stromal cell system (Shamash et al, 1994)

Seven low-grade B-cell lymphomas, five centroblastic/centrocytic and follicular, one small-cell lymphocytic, one lymphoplasmacytoid, according to the updated Kiel Classification [five follicle centre follicular, two small-cell lymphocytic according to the Revised European American Lymphoma Classification (REAL)] (Harris et al, 1994), were investigated.

Fresh tissue was taken at biopsy and dissected in a medium of RPMI 2% with 5% FCS, Hepes 25 mmol $l^{-1}$ (Gibco) and gentamicin 50 μg $ml^{-1}$ (Roussel Laboratories, Uxbridge, UK). The suspension was centrifuged on Ficoll Hypaque at 600 ***g*** for 25 min at 4°C to isolate the mononuclear fraction. The resting cells were washed twice in the medium described above before being frozen at a concentration of $2 \times 10^7$ $ml^{-1}$ in a freezing mixture of RPMI 2% with 20% FCS, 10% dimethylsulphoxide (Fluka) and stored in liquid nitrogen. The cells were resuspended in 2% RPMI with 10% FCS and penicillin/streptomycin and glutamine. Viability was assessed by 0.2% trypan blue exclusion. This was greater than 80% in all samples studied.

### *Cell culture*

Mouse fibroblasts (Ltk cells) transfected with the CD32 Fc receptor (obtained from the American Tissue Culture Collection with the permission of Dr KW Moore) were grown to confluence in 24-well culture plates (Becton Dickinson), in 2% RPMI supplemented with glutamine and 10% FCS and HAT (Sigma) and then irradiated to 75 Gy. Seven lymphoma cell suspensions were thawed from liquid nitrogen. The medium was aspirated and the lymphoma cells were suspended at a concentration of $5 \times 10^5$ cells $ml^{-1}$, 2 ml per well in 24-well plates (Becton Dickinson) in a growth medium consisting of Iscove's modified Dulbecco's medium without FCS (Gibco) with 50 mg $l^{-1}$ human holotransferrin; bovine serum albumin 5 g $l^{-1}$; bovine insulin 5 mg $l^{-1}$ long-chain fatty acids (*cis*-9-octadecenoic, all *cis*-9, 12-octadecadienoic and hexadecanoic acid all at 1 mg $l^{-1}$) (all from Sigma), FCS 2% (v/v), penicillin and streptomycin (Gibco), gentamicin 40 mg $l^{-1}$ (Roussel) and amphotericin 2.5 mg $l^{-1}$ (Squibb), anti-CD40 1 mg $l^{-1}$ (Seretec), interleukin 3 (IL-3) 20 μg $l^{-1}$ and interleukin 10 (IL-10) 100 μg $l^{-1}$ (both kindly provided by Amgen Biologicals, Thousand Oaks, CA, USA). Cells were grown for 8–10 days and monoclonal expansion confirmed using light chain restriction as assessed by flow cytometry. The percentage of contaminating T cells was assessed using CD3 FITC. Four wells were treated with each drug, or drug combination (dantrolene, 4-hydroperoxycyclophosphamide, etc., four wells acted as the untreated control, four wells as a control for dantrolene containing its diluent only).

### Assessment of cell death

Cell death was assessed using 0.2% trypan blue exclusion. Cells were counted using a haemocytometer.

### Preparation of drugs

Dantrolene sodium (kindly provided by Roger Phipps, Proctor and Gamble Pharmaceuticals, NY, USA, who advised on drug preparation). For cell line work, dantrolene was dissolved in glycerol 2.5 mg $ml^{-1}$ (6 mmol $l^{-1}$), sonicated and stored in the dark. For primary lymphoma culture, it was prepared in aqueous solution; 50 mg was dissolved in 75 ml of distilled water, to this was added 5 g of mannitol (Sigma). The mixture was sonicated for 2 min and then 4 mg of sodium hydroxide was added before storing in the dark for a maximum of 48 h (1.675 mmol $l^{-1}$).

Azumolene was kindly provided by Proctor and Gamble Pharmaceuticals, NY, USA in the form of a water-soluble white powder (9.06 mg $ml^{-1}$, 20 mmol $l^{-1}$).

Nimodipine (kindly provided by Bayer). This was prepared by dissolving 4.18 mg of crystalline powder in 10 ml of ethanol and storing away from light (1 mmol $l^{-1}$).

The following concentrations were studied: azumolene (A) 20, 10 and 5 μmol $l^{-1}$; dantrolene (D) 60, 30 and 15 μmol $l^{-1}$; nimodipine (N) 1, 0.5, 0.25 μmol $l^{-1}$ and combinations: D 60 μmol $l^{-1}$ and N 1 μmol $l^{-1}$; D 30 μmol $l^{-1}$ and N 0.5 μmol $l^{-1}$; and D 15 μmol $l^{-1}$ and N 0.25 μmol $l^{-1}$. The concentrations were chosen because 40–50 μmol $l^{-1}$ dantrolene represents the maximum plasma concentration achieved in humans and 0.7 μmol $l^{-1}$ nimodipine – the therapeutic plasma concentration. Nimodipine was chosen rather than verapamil as fatal ventricular fibrillation has been described in animal experiments when the latter has been combined with dantrolene.

The range of doses of azumolene was selected by starting with the therapeutic concentrations that had been used in muscle contraction experiments (5 μmol $l^{-1}$) in anaesthetized rats (Dillon et al, 1992).

The following cytotoxic drugs were used: dexamethasone (3.4 mg $ml^{-1}$), vincristine (1 mg $ml^{-1}$) (as solutions from David Bull Pharmaceuticals), doxorubicin (2 mg $ml^{-1}$) (from Pharmacia) and 4-hydroperoxycyclophosphamide (4HC) (3 mg $ml^{-1}$) dissolved in ethanol. The cytotoxic drugs were incubated at increasing concentrations, the lowest concentration that resulted in >25% cell death at 48 h in the $DOHH_2$ cell line was chosen for studies in primary lymphoma culture.

Cells were grown from five low-grade lymphomas (L211, L304, L503, L1103 and L1203) for 48–72 h. At this point, dantrolene (30 or 60 μmol $l^{-1}$) or 4HC (10 μmol $l^{-1}$) or the combination 4HC (10 μmol $l^{-1}$), vincristine (10 μmol $l^{-1}$), doxorubicin (0.1 mmol $l^{-1}$) and dexamethasone (10 μmol $l^{-1}$) were added. The four-drug regimen was chosen because it represented probably the most commonly used treatment programme for lymphoma (CHOP: cyclophosphamide, doxorubicin, vincristine and prednisolone). 4HC was used alone as it represented the alkylating drug cyclophosphamide, which is used as a single agent to treat low-grade lymphoma. The cells were analysed 120 h later.

### Assessment of P-glycoprotein-mediated drug efflux

Ability to excrete the dye $DIOC_2$ was used as a measure of P-glycoprotein drug efflux. It was tested in both the $DOHH_2$ cell line, the primary lymphoma and normal peripheral blood lymphocytes as controls (Chaudhary et al, 1992). Cyclosporin A (CsA) at a concentration of 2 μmol $l^{-1}$ was used to inhibit P-glycoprotein function as a positive control.

© Cancer Research Campaign 1998 

*Isolation of blood lymphocytes*

Twenty millilitres of blood was taken from a healthy volunteer into a 50 ml Falcon tube (BD) containing 50 µl of heparin (Monoparin 5000 iu $ml^{-1}$). An equal volume of RPMI 1640 was added to the tube and mixed by inversion. The diluted blood was divided and layered on 10 ml of Ficoll Hypaque (Nicomed, Oslo, Norway) and centrifuged at 600 ***g*** for 25 min at 25°C. After centrifugation, the mononuclear cell layer was carefully removed and the cells washed twice in RPMI.

*Lymphocyte dye loading and efflux*

Separated lymphocytes were resuspended in 10 ml of RPMI for 30 min with 6 µl of $DIOC_2$ (100 µg $ml^{-1}$) (Molecular Probe, Eugene, OR, USA) (final concentration 0.06 µg $ml^{-1}$). Cells were then washed twice at 4°C to remove extracellular dye and incubated at 37°C for 3 h to permit dye efflux. After incubation, they were washed with ice-cold media (including centrifugation at 4°C) and the remaining intracellular fluorescence measured by FACS analysis. Efflux was expressed relative to cells incubated at 4°C (instead of 37°C) or cells incubated with cyclosporin, both processes inhibiting dye efflux.

### Growth fraction

Cells were fixed in 70% ethanol. Ribonuclease type I (Boehringer Mannheim) 100 kilounits $ml^{-1}$ and then propidium iodide 50 µg $ml^{-1}$ were added. Propidium iodide fluorescence was detected at over 580 nm. Cells were gated using forward and light scatter; 5000 cells were analysed. Cells with propidium iodide fluorescence in the hypodiploid and diploid $G_0/G_1$ peak and the hyperdiploid S + $G_2$ (growth fraction) were determined on red fluorescence histograms using Lysys II software.

### Assessment of Bcl-2 expression by FACS analysis

As this oncoprotein is found in various sites within cells, permeabilization of the membrane is required. The cells were suspended in 0.3% Saponin (Sigma) in phosphate-buffered saline (PBSA) for 15 min at 25°C. They were then phenotyped using anti-bcl-2 FITC (Dako) (10 µl $10^{-6}$ cells per 100 µl) in 0.1% saponin solution for 30 min at 25°C. They were then centrifuged and resuspended in PBSA with 1% paraformaldehyde. The negative control (IgG1 mouse FITC, Dako) was similarly prepared. Expression was described in the form of mean channel fluorescence vs the control.

**Figure 1** Effect of different concentrations of dantrolene in the $DOHH_2$ cell line

### Assessment of dantrolene uptake

This was carried out using flow cytometry, as dantrolene autofluoresces in the FL2 band and previous studies have suggested that autofluorescence may be an indicator of uptake (Flewellen et al, 1993).

### Calculation of viable cell count in primary lymphoma culture

Four wells in each cultured lymphoma were exposed to each drug or drugs in combination, or in the case of the controls to the drug diluents alone. The wells were then aspirated, and the cells pooled together. The number of viable cells in each group was calculated by multiplying the total by the percentage viability as assessed by exclusion of 0.2% trypan blue; this also compared to the untreated control group.

### Reproducibility of results and statistical analyses

All cell line work was carried out in duplicate. The primary lymphoma culture results were single experiments. In each lymphoma, a drug was applied to four wells of the 24-well plate and the cells combined from the four wells for analysis.

Different schedules and doses were analysed by a balanced multiple analysis of variance (Minitab version 10, OH, USA). The residuals from this analysis were tested to confirm normality of distribution. This was the case in all ($P > 0.1$) except for nimodipine for which $P = 0.01$. However, as there was clearly no effect with nimodipine, a non-parametric analysis was not performed.

### RESULTS

### $DOHH_2$ cell line

*Prolonged culture of $DOHH_2$ cells with dantrolene and/or nimodipine (Figure 1)*

The total viable cell count was analysed at 72 and 156 h. Cell death was progressive, with a fall in cell numbers apparent by 72 h for dantrolene compared with the control culture. Only at a high concentration of 60 µmol $l^{-1}$ were cell numbers actually reduced compared with the count at time 0. In the lower concentrations, a retarding effect on cell growth was seen, the numbers rising more slowly than control (balanced analysis of variance of number of viable cells in control, 15 µM and 30 µM dantrolene cultures, controlling for dose and time, $n = 3$, $P < 0.001$). No evidence of activity for nimodipine alone was observed ($P = 0.645$), with control culture cell count at 156 h = 38.0 ± 3.0 × $10^5$ $ml^{-1}$, dantrolene 60 µmol $l^{-1}$ = 3.0 ± 1.0 × $10^5$ $ml^{-1}$, dantrolene 60 µmol $l^{-1}$ and nimodipine 1 µmol $l^{-1}$ = 3.5 ± 0.8 × $10^5$ $ml^{-1}$ and nimodipine 1 µmol $l^{-1}$ alone = 36.0 ± 7.5 × $10^5$ $ml^{-1}$. At 156 h the cytotoxic effects of dantrolene alone matched those of the combination of dantrolene and nimodipine at all concentrations studied ($P = 0.859$ by analysis of variance).

*Comparison of azumolene with dantrolene (see Figure 2)*

Cells were incubated for 168 h with three concentrations of dantrolene (20, 40 and 60 µmol $l^{-1}$) and three concentrations of

© Cancer Research Campaign 1998

**Figure 2** Comparison of single-agent activity of dantrolene and azumolene in the DOHH$_2$ cell line assessed at 168 h

**Figure 3** Mean results of primary lymphoma cultures – dantrolene vs 4HC vs 'CHOP'. Percentage of viable cells vs untreated control

**Table 1** Assessment of P-glycoprotein efflux using D1CO$_2$ fluorescent dye excretion in culture lymphoma cells with benign peripheral blood lymphocytes as controls

| Lymphomas | Percentage of bright cells (retaining fluorescent dye) 0 µmol CSA | 2 µmol CSA |
|---|---|---|
| L304 | 54.8 | 59.3 |
| L503 | 81.3 | 81.4 |
| L1103 | 91.2 | 66.2 |
| L1203 | 59.6 | 70.1 |
| Peripheral blood lymphocyte control | 28.5 | 93.9 |

azumolene (5, 10 and 20 µmol l$^{-1}$). The maximal efficacy of the two drugs appeared similar with azumolene appearing three to four times more potent than dantrolene.

### *Assessment of multidrug resistance – P-glycoprotein using DIOC$_2$ fluorescent dye extraction*

### ***Benign peripheral blood lymphocytes (controls)***

Lymphocyte control cells, incubated at 0°C, had a mean cellular fluorescence of 2470 units (range 858–9900). Markers were set about fluorescence distribution for cells incubated at 0°C and, for all other incubations, the proportion of cells falling within these markers was calculated (bright cells).

Across the concentration range for both dantrolene (0.5–20 µmol l$^{-1}$) and nimodipine (0.5–1 µmol l$^{-1}$) peripheral blood lymphocytes showed no difference in drug efflux compared with control (28–31% cells bright), i.e. not effluxing fluorescent dye. Higher concentrations of dantrolene could not be assessed using this method as the compound autofluoresces.

The addition of CsA (an inhibitor of P-glycoprotein) increased the number of bright cells from 31% to 81% demonstrating the presence of an active P-glycoprotein pump that was clearly not affected by dantrolene or nimodipine.

### ***DOHH$_2$ cell line***

Of the cells, 95% were bright in the absence of cyclosporin and 85% in the presence of cyclosporin (2 µmol l$^{-1}$), suggesting the absence of a functional P-glycoprotein system. No effect was seen with the addition of nimodipine 0–1 µmol l$^{-1}$. The autofluorescence of dantrolene meant that its effect could not be analysed using this method.

### Primary lymphoma culture

### *Assessment of multidrug resistance – P-glycoprotein using DIOC$_2$ fluorescent dye efflux*

In primary lymphoma cultures incubated with 0 and 2 µmol l$^{-1}$ cyclosporin for 2 h, the difference in bright cells was not significant

**Table 2** Lymphoma subtype, growth fraction and bcl-2 expression in primary culture compared with DOHH$_2$ cell line

| Lymphoma | Subtype (REAL classification) | Growth fraction Day 1 | Growth fraction Day 8 | Bcl-2 expression (percentage vs IgG$_1$ negative control) Day 1 | Bcl-2 expression Day 8 |
|---|---|---|---|---|---|
| L211 | Follicle centre, follicular | 7 | 7 | 97 | 36 |
| L304 | B-cell small lymphocytic | 3 | 2 | 0 | 93 |
| L503 | Follicle centre, follicular | 6 | 4 | 9 | 35 |
| L802 | Follicle centre, follicular | 10 | NA | 4 | NA |
| L903 | B-cell small lymphocytic | 7 | 6 | 92 | 89 |
| L1103 | Follicle centre, follicular | 18 | 23 | 47 | 80 |
| L1203 | Follicle centre, follicular | 10 | 11 | 92 | 6 |
| DOHH$_2$ cell line | Transformed follicle centre lymphoma | 40 | 40 | 34 | 34 |

NA, not available.

*© Cancer Research Campaign 1998*

**Table 3** Percentages of viabilities using trypan blue exclusion and the percentage of total viable cells vs an untreated control in five primary low-grade lymphomas treated with dantrolene 30 μmol l-1 and 60 μmol l-1, 4HC and 'CHOP' chemotherapy

| | L211 | | L304 | | L503 | | L1103 | | L1203 | |
|---|---|---|---|---|---|---|---|---|---|---|
| | (V%) | (T%) | (V%) | (T%) | (V%) | (T%) | (V%) | (T%) | (V%) | (T%) |
| Control | 97 | 100 | 94 | 100 | 95 | 100 | 95 | 100 | 90 | 100 |
| D-60 | 76 | 44 | 80 | 42 | 79 | 47 | 81 | 70 | 79 | 64 |
| D-30 | 93 | 76 | 89 | 44 | 82 | 36 | 89 | 67 | 83 | 64 |
| 4HC-10 | 88 | 66 | 70 | 23 | 78 | 33 | 89 | 47 | 74 | 41 |
| 'CHOP' | 54 | 12 | 56 | 9 | 56 | 12 | 70 | 13 | 58 | 18 |

V%, percentage of viable cells in each group as shown by trypan blue exclusion; T%, total number of viable cells in each group shown as a percentage of total viable cells in control group. D-60, dantrolene 60 μmol l-1; D-30, dantrolene 30 μmol l-1; 4HC-10, 4HC 10 μmol l-1; 'CHOP', 4HC 10 μmol l-1; doxorubicin 0.1 μmol l-1; vincristine 1 μmol l-1; dexamethasone 10 μmol l-1.

[median 70.5% bright vs 68.2% (cyclosporin treated) $P = 1.0$ (Mann–Whitney)]. Individual results are presented in Table 1. The quality of cells meant that this experiment could only be carried out once.

This suggests that the lymphoma cells did not possess a functional P-glycoprotein system.

### *The effect of calcium channel blockade with dantrolene alone, 4HC or 'CHOP' chemotherapy in primary lymphoma cultured cells (Figure 3 and Table 2)*

The growth fraction (GF) of the control group was assessed at 8 days and compared with the initial GF of the biopsy material (Table 2). Bcl-2 expression is also shown and can be seen to vary between one tumour and another at the start and during culture. Flow cytometry confirmed light chain restriction and a B-cell proliferation by CD19 + CD22 staining, suggesting that the malignant cells were proliferating. The percentage of T cells remained stable during culture (data not shown). Dantrolene uptake into cells was confirmed by autofluorescence. Dantrolene at 30 and 60 μmol l-1 had similar effects, unlike in the cell line (Figure 3). Dantrolene appeared equivalent to single-agent 4HC and all appeared inferior to the four-drug regimen. A table showing the individual results (Table 3) with the percentage viability of cells counted in each treated group (V) is shown, as well as the total viable cell count shown as a percentage of the untreated control (T). The mean results of all five primary cultures showing the total viable cell count in each group as a percentage of the untreated control is shown in Figure 3.

### *Dantrolene vs azumolene in three primary lymphoma cultures (Figure 4)*

Three lymphoma cultures were set up as above and dantrolene 60 μmol l-1 or azumolene 6 μmol l-1 was added for 120 h before analysis. In the three samples studied, at these concentrations, the two drugs were equally efficacious.

### DISCUSSION

This study was designed to assess the activity of drugs that are known to affect calcium repartitioning – a process thought to be important in initiating apoptosis (Baffey et al, 1993). Two drugs, dantrolene and azumolene, appeared to have a direct cytotoxic effect, while the third, nimodipine, appeared not to. This effect was studied both in a cell line known to produce anti-apoptotic protein bcl-2 and in low-grade B-cell lymphoma primary cultures, in which bcl-2 is also present. The cytotoxic activity of dantrolene and azumolene

**Figure 4** The effect of dantrolene vs that of azumolene in three lymphoma cultures assessed after 120 h continuous incubation appears independent of the level of bcl-2 in primary lymphoma culture initially, and indeed the level of bcl-2 varied during culture between the lymphomas studied; in addition, it appeared to be unrelated to the growth fraction. Whether the activity of these drugs is via disruption of this system or some other vital calcium-dependent pathway or indeed may be completely calcium independent remains an open question, as there is increasing evidence that absolute bcl-2 levels are not as important as the concentration relative to other members of the family, e.g. bax or bcl.

The results of the experiments with the $DOHH_2$ cell line show that dantrolene has a direct cytotoxic effect, whereas nimodipine either alone or in combination does not. A dose response to dantrolene is seen across the range of 15–60 μmol l-1. A cytostatic action is seen for 30 μmol l-1 with the 60 μmol l-1 being significantly more effective and cytotoxic. In the primary culture, however, 30 and 60 μmol l-1 appear equivalent.

Again, the addition of nimodipine appeared to add little. This may be because most cytosolic calcium changes depend on repartitioning between endoplasmic reticulum and cytosol rather than receiving calcium for the extracellular space (Charles et al, 1993). A similar calcium blocker, verapamil, has been reported to cause inhibition of B-cell proliferation in vitro and to induce clinical responses to B-cell chronic lymphocytic leukaemia (CLL) (Berrebi et al, 1994). It has been suggested that this might be due to tumour necrosis factor (TNF) release inhibition (Berrebi et al, 1994).

The concentration of dantrolene required for the observed effects is higher than in many studies of its actions. Its effects in muscle contraction depression, both in vitro and in vivo, appear to be

© Cancer Research Campaign 1998

maximal at the 10 μmol $l^{-1}$ range (Flewellen et al, 1993). Various experiments have used concentrations of up to 300 μmol $l^{-1}$ (Smith and Nahorski, 1993). Suppression of intracellular calcium mobilization needed to stimulate gluconeogenesis in rat hepatocytes has been achieved using 60 μmol $l^{-1}$ of dantrolene (Koike et al, 1992). Doses have been given to humans that should produce concentrations in the region of 40–50 μmol $l^{-1}$ without ill effect (Blank and Boggs, 1990). High concentrations administered to Beagle dogs using lyophilized dantrolene/mannitol and sodium hydroxide showed no effect of the mannitol sodium hydroxide and a 54% inhibition of skeletal muscle contraction in dantrolene doses producing levels of 55–60 μmol $l^{-1}$, with no effect on heart or blood pressure (Ellis et al, 1976). Such concentrations appear to be feasible for investigation.

Azumolene appeared to be a particularly interesting agent in that it was equipotent to dantrolene in skeletal contraction inhibition (Dhillon et al, 1992) but three to four times more potent in suppression of lymphoma proliferation in vitro. The efficacy of the two agents appeared equivalent.

Assessment of the multidrug resistance phenotype was considered important to rule out the explanation that any effect was simply due to blockade of the P-glycoprotein pump. In fact, there was no evidence that either the $DOHH_2$ cell line or the primary cultured cells studied had a functional P-glycoprotein system as assessed by $DIOC_2$ dye excretion. Indeed, even if they had, it would fail to explain the direct effect of dantrolene on the cell line and primary cultures.

The experiments comparing dantrolene to 4HC to a four-drug combination 'CHOP' in primary culture demonstrated dantrolene and 4HC to have equivalent effects. 4HC was chosen as it is a representative of the alkylator group of drugs that appear to be equivalent to multiagent treatment in vivo in previously untreated low-grade NHL and remain the standard first-line drugs for the treatment of low-grade lymphoma (Lister et al, 1978). Although the 'CHOP'-like four-drug regimen appeared more effective, this may simply be equivalent to the more rapid response to multidrug treatment compared with single agents seen in vivo (Horning et al, 1993) as analysis was carried out at 120 h, rather than over a much longer period when the results may have tended to converge.

In summary, unexpected single-agent activity of dantrolene and azumolene has been demonstrated in low-grade lymphoma, firstly in a cell line and more importantly in primary lymphoma culture. In primary culture, dantrolene appears equivalent to 4HC, which in this system and in vivo has consistent activity; of note, however, the effects are much less impressive than in the cell line. The role of dantrolene or azumolene as candidate therapies for low-grade lymphoma warrant further investigation.

### ACKNOWLEDGEMENTS

We thank Professor Peter Beverley, Middlesex Hospital, London, for his advice in the production of the paper; the Radiotherapy Department, St Bartholomew's Hospital, London, for fibroblast irradiation; and Mary Cotter for typing the manuscript.

### REFERENCES

Baffy G, Miyashita T, Williamson JR and Reed JC (1993) Apoptosis induced by withdrawal of interleukin 3 from an IL3 dependent hematopoietic cell line is associated with repartitioning of intracellular calcium and is blocked by enforced *bcl-2* oncoprotein production. *J Biol Chem* **268**: 6511–6519

Berrebi A, Shtalrid M, Klepfish A, Bassous L, Kushnir M, Shulman L, Vorst E and Hahn T (1994) Verapamil inhibits B cell proliferation and tumor necrosis factor release and induces a clinical response in B cell chronic lymphocytic leukemia. *Leukemia* **8**: 2214–2216

Berridge MJ (1993) A tale of two messengers. *Nature* **365**: 388–389

Blank JW and Boggs SD (1990) Successful treatment of an episode of malignant hyperthermia using a large dose of dantrolene. *J Clin Anesth* **5**: 69–71

Charles AC, Dirksen ER, Merril JE and Sanderson MJ (1993) Mechanisms of intracellular calcium signalling in glial cells studied with dantrolene and thapsigargin. *Glia* **7**: 134–145

Chaudhary PM, Macnetner EB and Roninson IB (1992) Expression and activity of the multidrug resistance P-glycoprotein on human peripheral blood lymphocytes. *Blood* **80**: 2735–2739

Dershwitz M. and Sreter EA (1990) Azumolene reverses episodes of malignant hyperthermia in susceptible swine. *Anesth Analg* **70**: 253–255

Dhillon DS, Pong SF and Moorehead TJ (1992) A comparison of the potency and specificity of the direct muscle relaxant activity of azumolene and dantrolene. *Drug Dev Res* **25**: 161–169

Ellis KO, Butterfield JL, Wessels FL and Carpenter JF (1976) A comparison of skeletal cardiac and smooth muscle actions of dantrolene sodium – a skeletal muscle relaxant. *Arch Int Pharmacodyn* **224**: 118–132

Erausquin G, Brooker G, Costa E and Wojcik WJ (1992) Stimulation of high affinity γ-amino butyric acid B receptors potentiates the depolarisation induced by intraneuronal ionised calcium content in cerebella granule neurons. *Mol Pharmacol* **42**: 407–414

Flewellen EH, Nelson TE, Jones WP, Arens JF and Wagner DL (1993) Dantrolene dose response in awake man: implications of management of malignant hyperthermia. *Anesthesiology* **59**: 275–280

Galione A (1993) Perspective: cyclic ADP-ribose: a new way to control calcium. *Science* **259**: 325–326

Garrido JJ, Arativetes RM, Iternanz A and Fuenta M (1992) Modulation by neurotensin and neuromedin N of adherence and chemotaxis capacity of murine lymphocytes. *Regul Pept* **41**: 27–37

Harris NL, Jaffe ES, Stein H, Banks PM, Chan JKC, Cleary ML, Delsol G, Wolf-Peters C, Falini B, Gatler KC, Grogan TM, Isaacson PG, Knowles DM, Mason DY, Muller-Hermelink H, Pileri S, Piris MA, Ralfkiaer E and Warnke RA (1994) A revised European-American classification of lymphoid neoplasms: a proposal from the International Lymphoma Study Group. *Blood* **84**: 1361–1392

Horning SJ (1993) Natural history of and therapy for the indolent non-Hodgkin's lymphomas. *Semin Oncol* **20**: 75–88

Katzung BG and Chatterjee K (1989) Vasodilators and the treatment of angina pectoris. In *Basic and Clinical Pharmacology*, 4th edn, Katzung BG. (ed) pp. 140–151. Prentice Hall: New York

Koike M, Kashiwagura T and Takeguchi N (1992) Gluconeogenesis stimulated by extracellular ATP is triggered by the initial increase in the intracellular $Ca^{2+}$ concentration of the periphery of hepatocytes. *Biochem J* **283**: 265–272

Lam M, Dubyak G, Chen L, Nunez G, Miesfeld RL and Distelhorst CW (1994) Evidence that *bcl-2* represses apoptosis by regulating endoplasmic reticulum associated calcium fluxes. *Proc Natl Acad Sci USA* **91**: 6569–6573

Leslie GC and Part NV (1989) The effect of EU4093 (azumolene sodium) on the contractor of intrafusal muscle in the soleus muscle of the anaesthetized rat. *Br J Pharmacol* **97**: 1151–1156

Lister TA, Cullen MH, Beard MEJ, Brearley RL, Whitehouse JMA, Wrigley PFM, Stansfeld AG, Sutcliffe SBJ, Malpas JS and Crowther D (1978) Comparison of combined and single agent chemotherapy in non-Hodgkin's lymphoma of favourable histological type. *Br Med J* **1**: 533–537

Miller RD (1989) Skeletal muscle relaxants. In *Basic and Clinical Pharmacology*, 4th edn, Katzung BG. (ed) pp. 323–333. Prentice Hall: New York

Seto S, Carrera CJ, Kubota M, Wasson BD and Carson DC (1985) Mechanism of toxicity of deoxyadenosine and 2-chlorodeoxyadenosine toxicity to non-dividing human lymphocytes. *J Clin Invest* **75**: 377–383

Shamash J, Norton AJ, Davies DC, Rohatiner AZS, Young BD and Lister TA (1994) A new stromal cell culture system for growing low grade B cell lymphomas using combined IL3 and IL10. *Blood* **84**: 446a

Smith SM and Nahorski SR (1993) Characterisation and distribution of inositol polyphosphate and ryanodine receptors in the rat brain. *J Neurochem* **60**: 1605–1614

Thorn P, Gerasimenko O and Petersen OH (1994) Cyclic ADP-ribose regulation of ryanodine receptors involved in agonist evoked cytosolic $Ca^{2+}$ ascillations in pancreatic alinar cells. *EMBO J* **13**: 2038–2043

Tian Q, Katz AM and Kim DH (1991) Effects of azumolene or doxorubicin induced $Ca^{2+}$ release from skeletal and cardiac muscle sarcoplasmic reticulum. *Biochim Biophys Acta* **1094**: 27–34

© Cancer Research Campaign 1998